# Novel molecular biological tools for the efficient expression of fungal lytic polysaccharide monooxygenases in *Pichia pastoris*

**DOI:** 10.1186/s13068-021-01971-5

**Published:** 2021-05-27

**Authors:** Lukas Rieder, Katharina Ebner, Anton Glieder, Morten Sørlie

**Affiliations:** 1 Faculty of Chemistry, Biotechnology, and Food Sciences, Norwegian University of Life Sciences (NMBU), Ås, Norway; 2 Bisy GmbH, Hofstätten a. d. Raab, Austria; 3 Institute of Molecular Biotechnology, Graz University of Technology, Petersgasse 14, Graz, Austria

**Keywords:** LPMO, *Pichia pastoris*, Signal peptide cleaving, Simplified expression

## Abstract

**Background:**

Lytic polysaccharide monooxygenases (LPMOs) are attracting large attention due their ability to degrade recalcitrant polysaccharides in biomass conversion and to perform powerful redox chemistry.

**Results:**

We have established a universal *Pichia pastoris* platform for the expression of fungal LPMOs using state-of-the-art recombination cloning and modern molecular biological tools to achieve high yields from shake-flask cultivation and simple tag-less single-step purification. Yields are very favorable with up to 42 mg per liter medium for four different LPMOs spanning three different families. Moreover, we report for the first time of a yeast-originating signal peptide from the dolichyl-diphosphooligosaccharide-protein glycosyltransferase subunit 1 (*OST1*) form *S. cerevisiae* efficiently secreting and successfully processes the N-terminus of LPMOs yielding in fully functional enzymes.

**Conclusion:**

The work demonstrates that the industrially most relevant expression host *P. pastoris* can be used to express fungal LPMOs from different families in high yields and inherent purity. The presented protocols are standardized and require little equipment with an additional advantage with short cultivation periods.

## Background

Lytic polysaccharide monooxygenases (LPMOs) are mono-copper-dependent oxidoreductases that catalyze the oxidative cleavage of glycosidic bonds of recalcitrant sugar polymers such as chitin, cellulose, and hemicelluloses [1–5]. LPMOs are proposed to be major players in the efficient enzymatic conversion of bio-based materials and have become a key ingredient in commercially available products for enzymatic saccharification. Their unique and powerful oxidative abilities make them promising candidates for the application in renewable energy technologies and interesting targets for enzyme engineering, especially in light of the large amount of carbohydrate-based waste [6–11].

Although the potential of LPMOs has been recognized a decade ago [1], research in this field still faces major challenges starting, most fundamentally, with the production of active enzymes. Since isolation from native hosts can be challenging due to low yields, secretion of enzyme cocktails, and cultivation obstacles, recombinant production in bacterial or eukaryotic model organisms is desired. Due to the extracellular biomass-degrading nature, all LPMOs are secreted from their native hosts, regardless of their origin being eukaryotic or prokaryotic, which facilitates secretion in the recombinant host systems and allows easier down-stream processing.

For the recombinant expression of LPMOs of eukaryotic origin, the yeast *Pichia pastoris* (*Komagataella phaffii*) is one of the more frequently used host systems [12]. Main reasons for the use of *P. pastoris* are typical eukaryotic post-translational modifications, its capacity to secrete recombinant proteins to a high titer, and the increasing number of tools available for efficient genetic engineering [13–15]. One of the main challenges in the recombinant expression of active LPMOs lies in the correct processing of the N-terminus, as all LPMOs share a highly conserved N-terminal histidine that is directly involved in the formation of the active site by coordinating the copper center with both the imidazole ring and the backbone N-terminal amine, the so-called *histidine brace* [16]. Thus, a correctly processed N-terminus is indispensable for the catalytic activity and highlights the importance of a well-tailored expression system for LPMOs as recently addressed in several publications [17–19].

For *P. pastoris* the most commonly used expression tools are the promoter of the *P. pastoris* alcohol oxidase 1 gene (P_*AOX1*_) in combination with the α-mating factor secretion signal of *Saccharomyces cerevisiae* that has successfully been applied for the recombinant expression of a multitude of proteins [20]. While the P_*AOX1*_, which requires induction by toxic methanol, has been used for the expression of LPMOs, the α-mating signal is not suitable for the recombinant expression of LPMOs [18]. Thus, the production of LPMOs in *P. pastoris* is dependent on native LPMO signal peptides and the hosts ability to recognize correctly foreign leading sequences of sometimes only distantly related organisms. While the problem of the signal peptide has been addressed for the expression of bacterial LPMOs in *E. coli* [21], no one has tackled the problem for eukaryotic expression systems.

In this study, we present a LPMO-tailored expression system that allows a streamlined and efficient state-of-the-art recombination cloning and easy protein expression with a subsequent one-step purification. As an alternative to native LPMO signal peptides, we present a yeast-originating signal peptide that facilitates secretion of the correctly processed LPMOs of fungal origin.

## Results and discussion

### Evaluation of the expression system, LPMO expression and purification

**Fig. 1:**
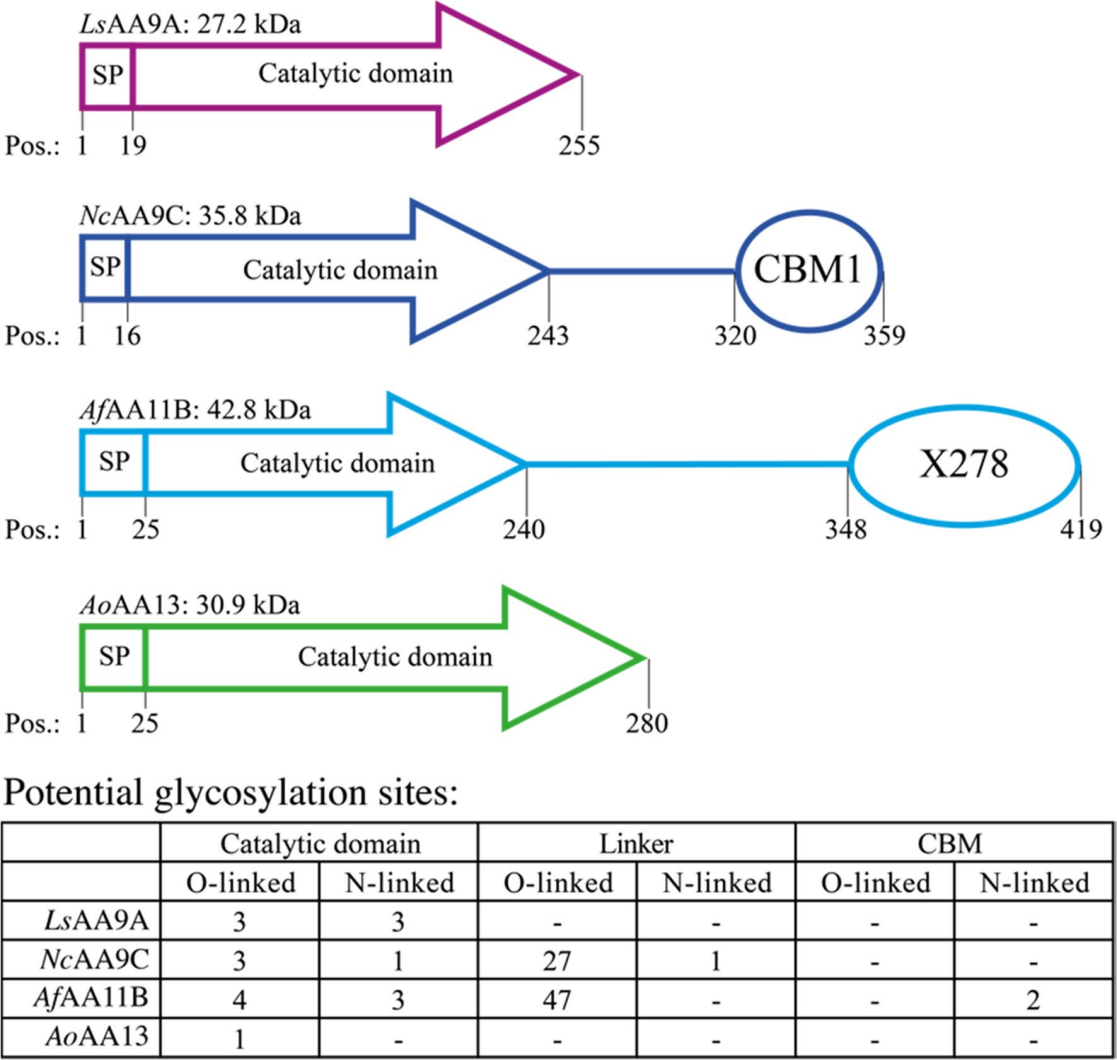
Schematic representation of the domain architecture of the LPMOs expressed in this study (top) including a table showing potential N- and O-linked glycosylation sites found on the catalytic domain, linker region and (potential) CBM (bottom) of the individual enzymes

We opted to use two well-described LPMOs of the auxiliary activity (AA) family 9 that have previously been expressed in *P. pastoris* [22, 23] to evaluate the applicability of the Bisy platform strain BSYBG11 (Bisy GmbH, Hofstätten a. d. Raab, Austria) in combination with the integrative *E. coli/P. pastoris* shuttle vectors pBSYP_*GCW14*_Z or pBSY3Z for the functional expression of LPMOs. The tested plasmids are almost identical but employ either the strong constitutive promoter of the uncharacterized *Chr1-4_0586* gene (P_*GCW14*_) [24, 25] or the 500-bp fragment of the derepressed promoter of the *CAT1* gene (P_*DC*_) [15] to control the transcription of the gene of interest (GOI). If not explicitly mentioned otherwise, the LPMOs were expressed with their native signal peptide (SP) to facilitate secretion to the extra-cellular environment. Despite belonging into the same family, the chosen genes originating from *Lentinus similis* (*Ls*AA9A) and *Neurospora crassa (Nc*AA9C) are encoding for LPMOs with a fundamental different domain architecture. *Ls*AA9A is a single-domain protein with only a catalytic domain whereas *Nc*AA9C is a multidomain protein with a catalytic domain and a CBM1 domain which is attached via a long threonine and serine-rich linker region (Fig. 1). Nonetheless, both genes were successfully cloned into the two tested plasmids and efficiently secreted by the *P. pastoris* BSYBG11 strain*.*

**Fig. 2:**
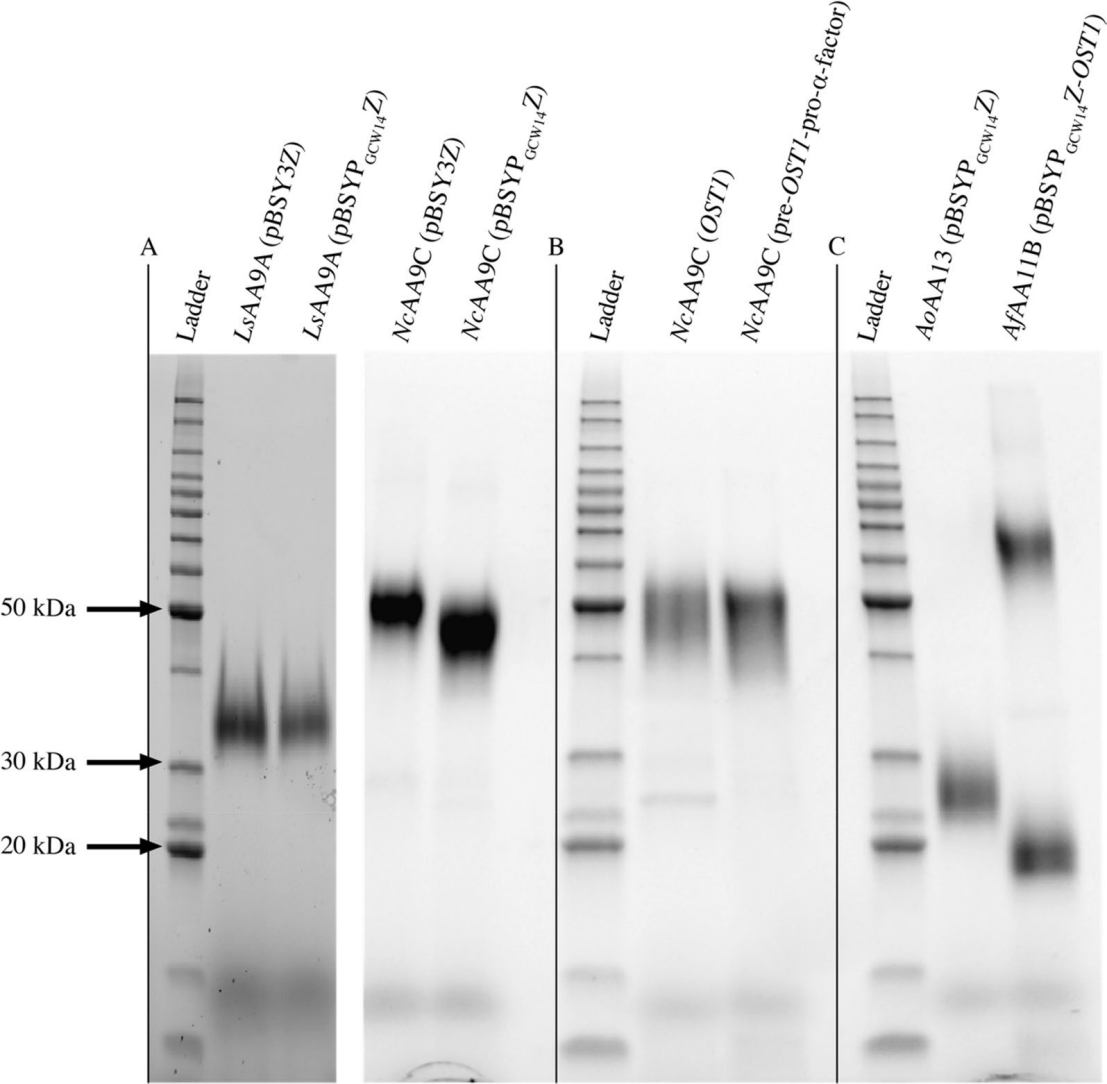
SDS-PAGE of the single-step purified enzymes (1 µg/well). **A** *Ls*AA9A and *Nc*AA9C expressed with either pBSY3Z or pBSYP_GCW14_Z and the native SP. **B** *Nc*AA9C expressed with either the *OST1* or the pre-*OST1*-pro-α-factor leading sequence in combination with the pBSYP_*GCW14*_Z plasmid. **C** *Ao*AA13 and *Af*AA11B expressed with the pBSYP_*GCW14*_Z plasmid in combination with the native and the *OST1* SP, respectively

Following transformation, selection, initial cultivation in micro-scale, and analysis based on titer of secreted protein, we chose the best producing clone for each construct for medium-scale enzyme production. This was performed in 500 ml YPD in 2-L baffled shake flasks and expression of the LPMOs was confirmed by SDS-PAGE. The predicted masses of *Ls*AA9A and *Nc*AA9C are 27.1 kDa and 35.8 kDa, respectively. In silico studies of the proteins via the online tools NetNGlyc and NetOGlyc (http://www.cbs.dtu.dk/services) indicated that the catalytic domain of both enzymes and the linker region of *Nc*AA9C are prone to be N- and O-glycosylated (Fig. 1). This prediction was confirmed by SDS-PAGE analysis showing the protein band of *Ls*AA9A at ~ 35 kDa and *Nc*AA9C at ~ 50 kDa, respectively (Fig. 2A).

After 60 h of cultivation, the total amount of secreted protein from 500 mL culture ranged from 21 to 76 mg for the different strains (Bradford assay). Interestingly, the strain carrying the pBSYP_*GCW14*_Z-*N*cAA9C construct showed significantly higher expression levels (76 ± 7 mg) compared to the other three selected expression strains (~ 25 mg). It has been noted that this clone carrying the pBSYP_*GCW14*_Z-*N*cAA9C construct was already an outlier in the initial screening and all other transformants carrying this construct showed significantly lower amounts of secreted protein. Therefore, the extraordinary performance of this strain is presumed to be due to integration effects (locus, copy number) of the expression cassette and not caused by regulatory features (promoter) or related to the GOI. Nonetheless, this observation stresses the importance of assessing the secretion capacity of the different transformants prior expression clone selection as multicopy integration and locus effects seem to be the key for an extraordinary LPMO production strain.

SDS-PAGE analysis of the expression supernatant displayed a clean secretome with mainly recombinant LPMO. Thus, we saw the opportunity to use a single-step purification approach using size exclusion chromatography (SEC) to keep purification fast and efficient. For this purpose, 10 ml of the fivefold concentrate, corresponding to 2–7 mg of protein, were concentrated, and loaded onto SEC column. The chromatograms obtained from the SEC purification revealed that *Nc*AA9C and *Ls*AA9A elute in single peaks corresponding to their mass difference at 50 and 60 ml, respectively. Analysis by SDS-PAGE showed a homogenous appearance of the purified enzyme solution underlying the power of this one-step purification system (Fig. 2A).

The performance of the strains was evaluated by assessing the yield of pure protein we could obtain from the one-step SEC purification. From 50 mL cultivation broth, which was concentrated prior to loading onto the SEC column, 0.61 ± 0.01 and 0.60 ± 0.03 mg of pure *Ls*AA9A and 0.39 ± 0.02 and 2.08 ± 0.06 mg pure *Nc*AA9C could be achieved with pBSY3Z and the pBSYP_*GCW14*_Z, respectively (Table 1). The shown yields correspond to 8—42 mg of pure LPMO per liter culture supernatant.

**Table 1:** Overview on the protein concentration (mg/ml) and total amount of protein (mg) of each step along the purification process of *Ls*AA9A and *Nc*AA9C expressed with either the pBSY3Z or the pBSY_*PGCW14*_Z expression plasmid and the native LPMO SP, from the crude supernatant to the pure protein

Enzyme	Plasmid + SP	Supernatant		Fivefold concentrated		Purified (obtained from 50 ml cultivation supernatant)		Recovered protein
		mg	mg/ml	mg	mg/ml	mg	mg/ml	%
*Ls*AA9A	pBSY3Z + native	21 ± 5	0.042 ± 0.010	22.5 ± 0.4	0.225 ± 0.004	0.61 ± 0.01	1.2 ± 0.02	27
	pBSYP_*GCW14*_Z + native	28 ± 2	0.056 ± 0.004	24.7 ± 1.7	0.247 ± 0.017	0.6 ± 0.03	1.4 ± 0.05	24
*Nc*AA9C	pBSY3Z + native	22.5 ± 1.5	0.045 ± 0.003	20.7 ± 2.3	0.207 ± 0.023	0.39 ± 0.02	0.8 ± 0.05	18
	pBSYP_*GCW14*_Z + native	76 ± 7	0.153 ± 0.014	70.5 ± 0.5	0.705 ± 0.005	2.08 ± 0.06	2.9 ± 0.03	29

For precise measurements, the volume of the supernatant and the fivefold concentrate was adjusted to 500 ml and 100 ml, respectively. Please note that the values reported in the column labeled with “purified” were obtained from 50 ml cultivation supernatant

Since it was the aim to make the production of LPMOs efficient it is interesting to compare the yields obtained with the here presented expression platforms to yields reported in literature. It appears the most frequently used regulatory sequences for the transcription of recombinant LPMO in *P. pastoris* are the strong methanol-dependent promoter of the alcohol oxidase 1 gene (P_*AOX1*_) or the strong constitutive glyceraldehyde-3-phosphate dehydrogenase promotor (P_*GAP*_) in combination with the native LPMO SP. Unfortunately, most publications that describe LPMO characterization using *P. pastoris* as host system and medium-scale cultivation using shake flasks, do not provide any data about expression or purification yields [23, 26–28].

The few works stating LPMO production titers were usually done on bioreactor scale using defined medium, methanol induction and cultivation times far exceeding 60 h, which makes comparisons with our system difficult. These often tediously optimized advanced expression approaches result in much higher extra cellular protein titers (up to 3 g) per liter of cultivation media [22, 29, 30] and consequently also in a higher titer of LPMO, up to 0.79 g, per liter culture supernatant before purification [22].

Although we report lower total yields (up to 150 mg), it has be noted that cultivations were done in shake flasks in standard YPD over 60 h without any optimization of cultivation conditions or media, we presume much higher titers can be achieved with our strains on larger scale. Nonetheless, this system was tested on medium scale with simple cultivation techniques to make it applicable for a broad range of end users without the need for specialized equipment. Additionally, the here presented system employs regulatory sequences that circumvent the need for methanol as inducer of protein production and are superior to the so far reported systems either in strength (P_*GCW14*_) [25] or regulatory qualities (P_*DC*_) [15]. However, it has be noted, that for the P_*DC*_ to reach its full potential a longer cultivation time would be advised, since this promoter’s strength of separating culture growth and protein production was not realized to the fullest in this work.

To summarize, the herein presented expression system allows simplified protein production in YPD media without the necessity of induction which can be performed over a relatively short time without the need for specialized equipment or expertise, but at this scale its productivity is not comparable with an optimized bioreactor system. Therefore, we propose the most feasible comparison of productivity is done based on the amount of pure LPMO recovered from the total amount of secreted protein. Like for the expression several methods are described for the purification of LPMOs in literature.

One strategy for the purification of LPMOs is purification tags. For LPMO purification, the use of N-terminal tags is generally not possible due to the N-terminal histidine that is involved in the formation of the active site. Thus, a C-terminal His-tag is most commonly used for the production of LPMOs. This strategy has been shown to result in yields of ~ 500 mg pure protein per liter cultivation broth [30] which is much higher than the yields obtained with standard chromatography techniques (Table 2). Nonetheless, the use of His-tags is problematic as histidines can bind free metal ions that lead to complications during analysis of the enzyme action. Additionally, it has been shown that His-tag purification causes severe damage to the active site resulting a lower enzyme activity [30]. To avoid metal coordination by the tag, a cleavable version of the His-tag using the TEV protease was engineered and used by Kadowaki et al. [31]. However, this approach requires overnight incubation at room temperature and two purification rounds to separate tagged and untagged LPMOs and in the end the enzyme still contains an overhang at the C-terminus at the TEV cleavage site.

**Table 2:** Comparison of expression constructs, cultivation and purification methods and the thereof resulting yields of different LPMO production protocols found in literature

Enzyme	Plasmid	Promoter	Signal peptide	Cultivation method	Purification steps	Total protein per liter culture media prior purification	Total protein per liter culture media after purification	Reference
*Tr*Cel61A	pPpT4	AOX1	Native	2-L fed-batch reactor	N.A	> 400 mg	N.A	[18]
*Nc*AA9J	pPICZαA	AOX1	Native	7-L fed-batch reactor	Three chromatographic steps	1574 mg^a^	24 mg^a^	[22]
*Nc*AA9C	pPICZαA	AOX1	Native	7-L fed-batch reactor	Two chromatographic steps	2762 mg	67 mg^a^	[22]
*Nc*AA9F	pPICZαA	AOX1	Native	7-L fed-batch reactor	Three chromatographic steps	1818 mg^a^	2.4 mg^a^	[22]
*Nc*AA9E	pPICZαA	AOX1	Native	7-L fed-batch reactor	Three chromatographic steps	1327 mg^a^	2.2 mg^a^	[22]
*Nc*AA9C	pPICZαA	AOX1	Native	500-mL fed-batch reactor	Two chromatographic steps	1370 mg^a^	90 mg^a^	[29]
AaAA16	pPICZαA	AOX1	Native	1.3-L fed-batch reactor	IMAC^b^, IEC	N.A	500 mg after IMAC	[30]
Several AA9s	pPICZT	AOX1	Native	2-L shake flask	IMAC	34 mg per 100 g cell wet weight	N.A	[31]

^a^Yield was adjusted to 1 L culture supernatant

^b^Resulted in LPMO inactivation

Another possibility to purify LPMOs that helps to avoid the earlier mentioned complications, is the use of standard chromatography approaches as outlined by Kittl et al. and Sygmund et al. [22, 29]. Depending on the enzyme they report two or three chromatography steps to obtain the pure LPMO which results in low enzyme recovery rates (< 8%) and final protein yields of 2–90 mg from bioreactor cultivation. Interestingly, the herein presented expression and purification strategy results in higher protein recovery rates (< 25%) and, as we think, in very competitive yields of pure LPMO (8–42 mg) keeping in mind the much simpler shake-flask cultivation.

### Substrate oxidation

**Fig. 3:**
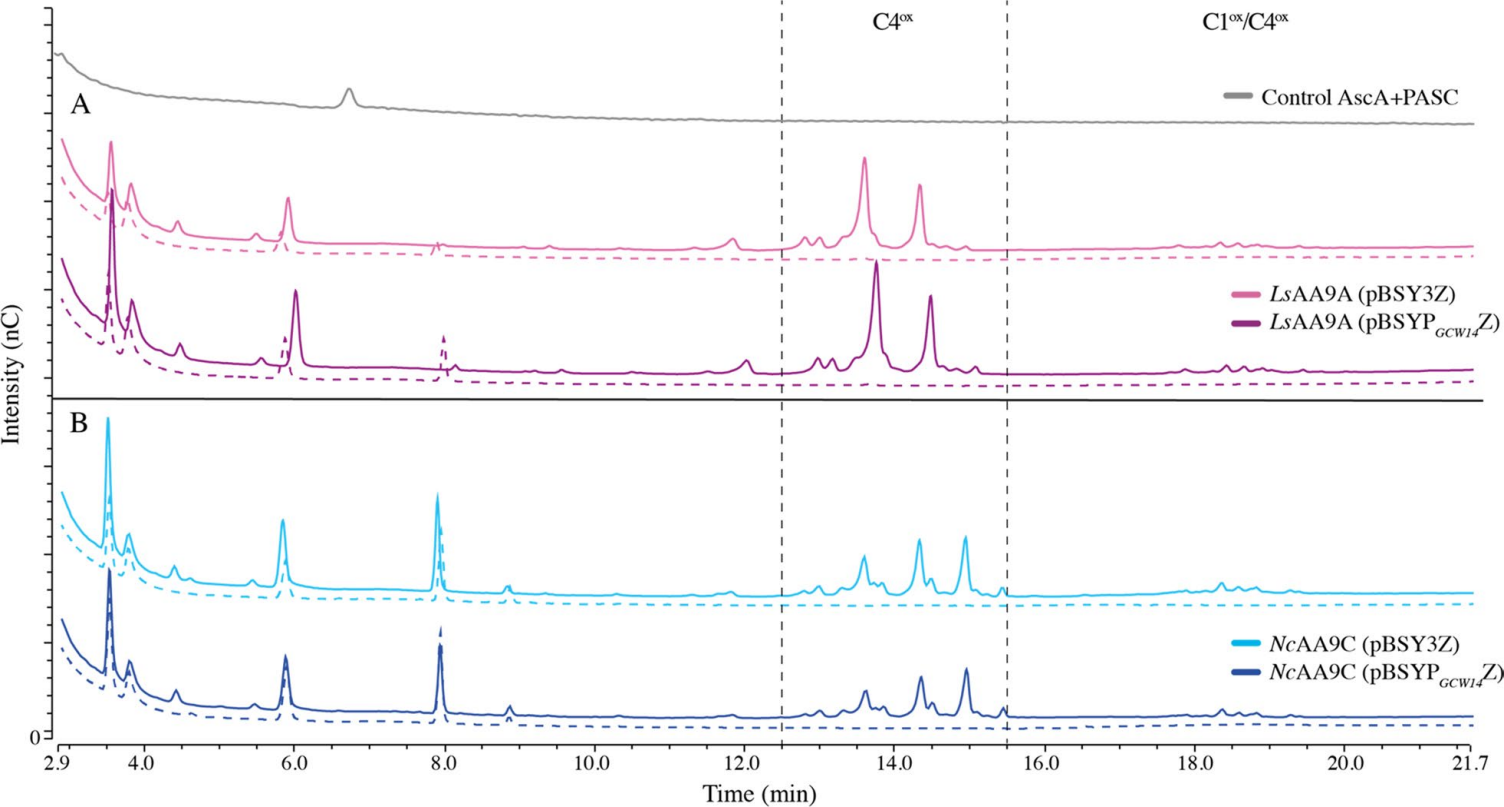
HPAEC-PAD profiles showing the products of the degradation of 0.1% PASC by 5 µM LPMO in aerobic conditions in the presence (solid lines) and absence (dashed lines) of 1 mM AscA. As catalyst either *Ls*AA9A (**A**, magenta) or *Nc*AA9C (**B**, blue) which were produced with the pBSY3Z (light color) or the pBSYP_*GCW14*_Z (darker color) expression plasmids and their native SP, were used

To assess LPMO catalysis, we incubated 5 µM of the enzymes, produced either with pBSY3Z (light color) or the pBSYP_*GCW14*_Z (dark color), with 0.1% PASC in the presence (solid lines) and absence (dashed lines) of 1 mM L-ascorbic acid (AscA) over night (Fig. 3). Analysis via HPAEC-PAD chromatography confirmed the oxidation of PASC by *Ls*AA9A and *Nc*AA9C in the presence of reductant shown through the formation of C4 and C1/C4-oxidized products in accordance with previous published studies [32, 33]. Furthermore, the identical product profiles confirm that the expression vectors and therefore the differently regulated LPMO transcription and expression in different growth phases, has no influence on the mode of action of the enzymes.

Moreover, the results equals those by Frandsen et al. showing that *Ls*AA9A does not require the methylation at the N-terminal histidine, which occurs upon expression in higher eukaryotic fungal expression hosts (e.g., *Aspergillus* species [16]) but not in *P. pastoris*, to perform substrate oxidation [23]. This finding however is not surprising as the posttranslational methylation is proposed to have no direct influence on the catalytic activity and is solely related to enzyme stability [26].

Interestingly, the results demonstrate the differences in activity between the two LPMOs as can be seen in the C4-oxidized product area of the chromatograms between 12- and 16-min retention time. Whereas *Nc*AA9C releases products ranging from DP2^ox^-DP4^ox^, *Ls*AA9A produces mainly DP2^ox^ and DP3^ox^, which demonstrates the ability of *Ls*AA9A to oxidatively cleave small oligomers in accordance with the study of Frandsen et al*.* [33].

### Hydrolytic background activity and product profile

**Fig. 4:**
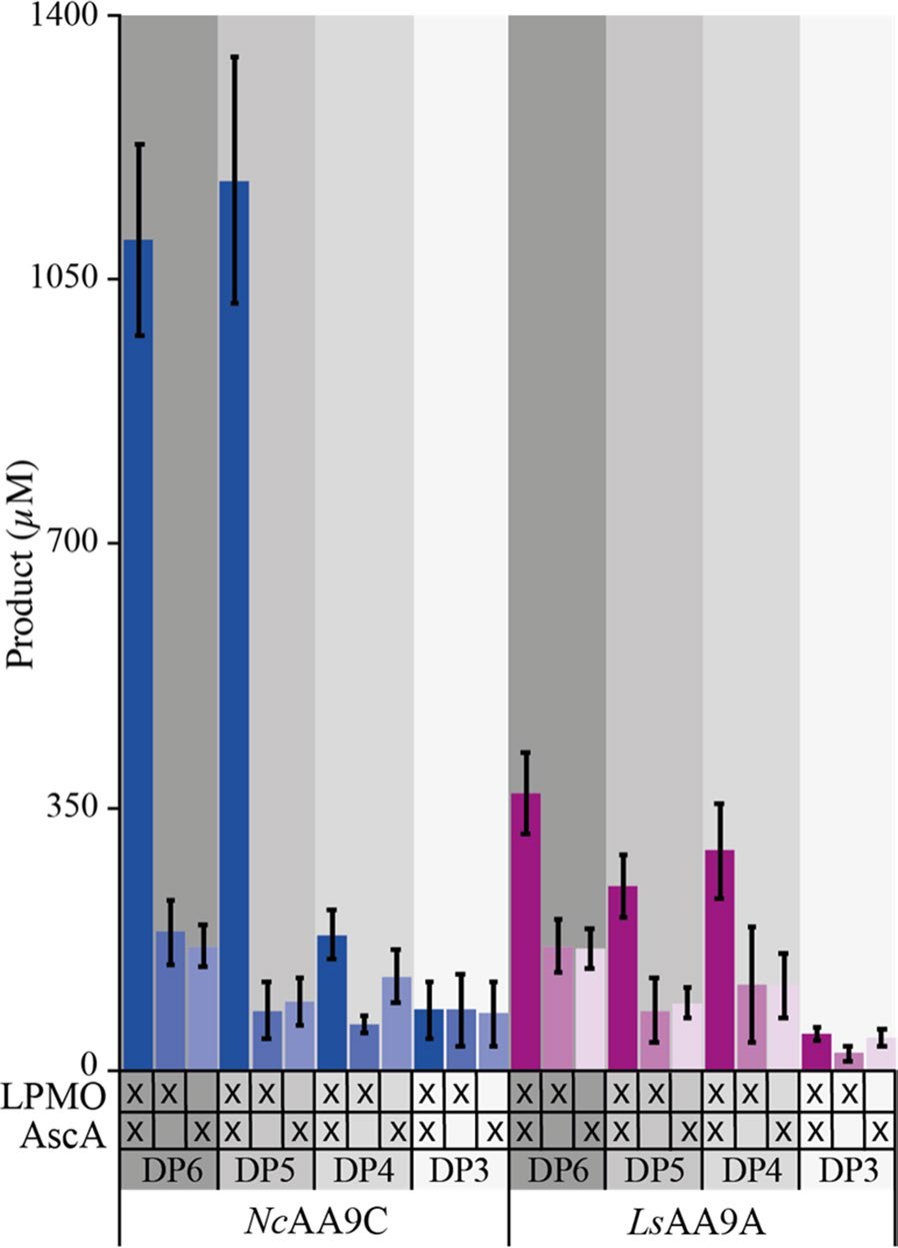
Oxidation of soluble cellulosic substrates with different degree of polymerization (DP3–DP6) by *Nc*AA9C (blue) and *Ls*AA9A (magenta) expressed with the pBSY_*GCW14*_Z and the native LPMO SP. Reactions were carried out in aerobic conditions and contained 1 µM LPMO, 1 mM oligomeric substrate and 1 mM AscA. Control reactions did either not contain reductant or LPMO which is indicated by the X bellow the bars showing the product concentrations. The presented numbers are the average of three biological replicates

The HPLC analysis shows the presence of native (non-oxidized) products in the absence of the reductant needed to initiate LPMO catalysis (Fig. 3). We wanted to confirm that product formation (native and oxidized) derives directly from LPMO catalysis and not from minor concentrations of endogenous secreted hydrolases that could not be removed by SEC. The following study was only done with enzymes produced with pBSYP_*GCW14*_Z plasmid, since previous analysis showed that LPMO quality was independent of the expression vector. Reactions were set up with 1 mM oligomeric substrates (DP3-6) in the presence of i) 1 µM LPMO and 1 mM reductant, ii) 1 µM LPMO and no reductant and iii) 1 mM reductant and no LPMO and incubated for 6 h prior quenching and analysis via HPAEC-PAD chromatography For product quantification we prepared standard curves for each oligomer ranging from DP2-DP6 and used linear regression to calculate the concentration of each native oligomer that was released from LPMO catalysis as they would increase proportionally with the oxidized products. The total amount of released product was obtained by summing up the concentration found for each oligomer (Fig. 4).

The results displayed in Fig. 4 confirm that only the presence of LPMO and reductant lead to the formation of product in the form of shorter oligomers. In both control reactions which were either without reductant or enzyme (experiments ii and iii), similar product levels were detected which would not be the case if the enzyme preparation would contain cellulases as they are reductant independent. Thus, the detected shorter oligomers most certainly originate from the purchased substrate which is in accordance with the > 95% purity pledged by the producer. Comparing the amount of released product after 6 h, it appears that *Nc*AA9C outperforms *Ls*AA9A on the oxidation of oligomers with DP6 and DP5. However, *Ls*AA9A has higher activity with DP4 compared to *Nc*AA9C. This confirms the observations made in the experiments with PASC that indicate that *Ls*AA9A is able to bind and oxidize shorter oligomers. These findings are in line with previous studies of *Nc*AA9C and *Ls*AA9A that showed the oxidation of oligomeric carbohydrates and indicated that *Ls*AA9A is active on shorter oligomers than *Nc*AA9C [23, 32].

### A new signal peptide for the secretion of active recombinant LPMOs

In 2014, Fitzgerald and Glick were the first ones to use the signal peptide of the dolichyl-diphosphooligosaccharide-protein glycosyltransferase subunit 1 (*OST1*) from *S. cerevisiae* for secretion of a model protein in *P. pastoris* [34]. Four years later, Barrero et al. created an artificial hybrid secretion signal the pre-*OST1*-pro-α-factor, which combines the pre-*OST1* sequence and the pro-region of the α-mating factor, they were able to successfully secrete a tetrameric far-red fluorescent protein (E2-Crimson) [35]. In contrast to most cleavable signal peptides the pre-*OST1* facilitates co-translational translocation to the ER as a way for mature proteins to enter the secretion pathway (opposed to post-translational translocation promoted for example by the pre-pro-α-mating factor) which can be beneficial for the secretion of aggregation-prone proteins [34].

Trying to simplify and improve recombinant LPMO expression, we not only targeted transcriptional regulation by different promoters, but also the topic of heterologous signal peptides for the secretion of active recombinant LPMOs. Since it is known that the most frequently used heterologous signal peptide for protein secretion with *P. pastoris*, the pre-pro-α-mating factor of *S. cerevisiae*, does not yield active enzyme [18] we searched for suitable alternatives that have previously achieved satisfying titers secreting recombinant proteins with *P. pastoris*. Therefore, we assessed the applicability of the pre-*OST1* signal peptide and its hybrid, for the expression of active LPMO. For this purpose, the native signal peptide of *Nc*AA9C was exchanged against the *OST1* and the pre-*OST1*-pro-α-factor and were used for protein production with *P. pastoris*. Expression and secretion could be confirmed for both LPMO-signal peptide constructs by SDS-PAGE (Fig. 2B). Single step purification from 50 mL broth yielded 1.38 ± 0.06 and 0.93 ± 0.1 mg of protein with *OST1* and the pre-*OST1*-pro-α-factor, respectively, which is less than the 2.08 ± 0.06 mg protein we obtained earlier with the same plasmid but with the native LPMO SP (Table 1). Nonetheless, it must be noted that it is not possible to directly compare the effect of the signal peptides with respect to obtained protein quantity as the most active clones of the landscape (clonal outliers) were chosen for LPMO production, which resulted in high protein titers, but brings the bias of locus and copy number effects [36, 37]. However, the data suggests that the expression of LPMOs is independent of the translocation process and therefore, the place of translation, as recombinant protein was obtained from both constructs.

To obtain active recombinant LPMOs, correct cleavage and processing of the enzymes N-terminus is essential since the conserved N-terminal histidine is part of the active site and plays an important role in the enzymes ability to catalyze substrate oxidation. To assess the applicability of the different recombinant fungal signal peptides to efficiently secret active functional LPMOs, HPAEC-PAD analysis was undertaken to determine oxidized products resulting from enzymes activity.

**Fig. 5:**
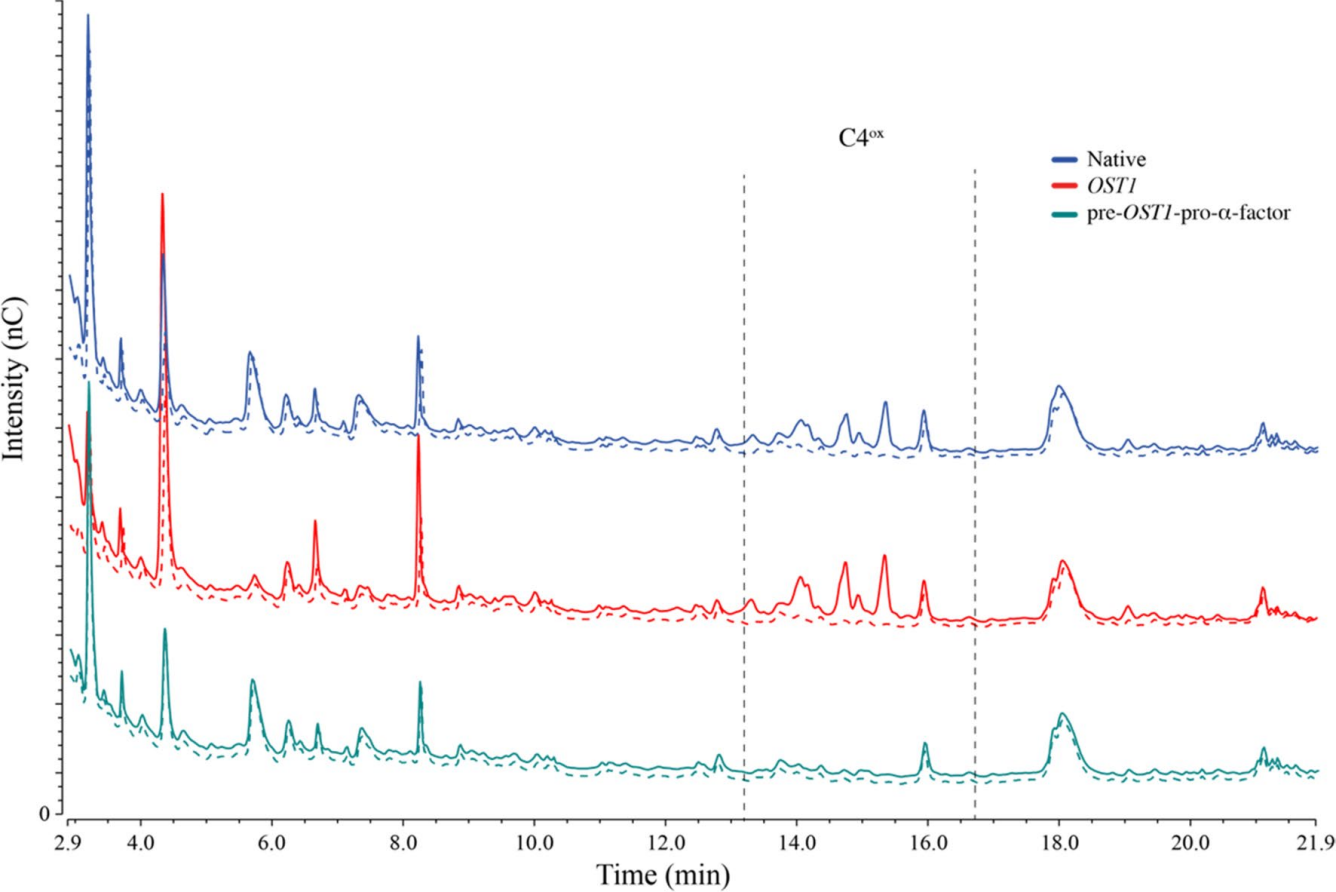
HPAC-PAD profiles of overnight reactions with 0.1% PASC and 50 µl of the fivefold unpurified, concentrated expression supernatant containing *Nc*AA9C expressed with different signal peptides in the presence (solid lines) and absence (dashed lines) of 1 mM AscA. The chromatograms in blue show the *Nc*AA9C expressed with the native signal peptide. The *OST1*-produced LPMO is shown in red and the reaction using the enzyme secreted with the pre-*OST1*-pro-α-factor is presented in green

Due to good expression levels, 50 µL of the fivefold concentrated, unpurified expression supernatant was directly incubated with 0.1% PASC in the presence (solid lines) and absence (dashed lines) of 1 mM AscA (Fig. 5). HPLC analyses clearly shows, that only reactions with *Nc*AA9C secreted with either the native or the *OST1* signal peptide contain C4-oxidized degradation products from PASC. In the reaction with the LPMO that was secreted with the pre-*OST1*- pro-α-factor, we could not observe any difference to the control reaction without reductant which implies that no substrate oxidation occurred indicating that the enzyme is inactive.

Moreover, regarding enzyme activity, two further observations were made when the concentrated culture supernatant was used as catalyst: firstly, no external CuSO_4_ had to be added to the reactions. This indicates that the enzyme naturally contains sufficient amounts of copper in their active site for reactivity, which is advantageous when thinking about industrial use of LPMOs or screening processes during enzyme engineering. Secondly, an external reductant was needed to obtain substrate oxidation. This suggests that no reductant powerful enough to prime the copper center is present once the cells were separated from the expression broth and the initial concentration step was performed which is highly beneficial as the likelihood of damage to the enzymes active center due to auto-oxidation during storage of the crude enzyme sample until purification is drastically reduced.

To confirm that inactivity of the LPMO secreted with the pre-*OST1*-pro-α-factor relates to incorrect processing of the N-terminus, a MALDI-ToF MS analysis was performed on trypsin digested LPMOs expressed with the different signal peptides. Based on in silico trypsin digestions (Table 5), a correctly processed N-terminus results in a peptide with the sequence: “**H**TIFQK”, having a mass of 773. Non-correctly processed N-termini would have increased masses due to additional amino acids N-terminal of the terminal histidine as the downstream located trypsin cleavage site would not be affected. The resulting peptides of proteins with incorrectly processed N-termini would have m/z values of 1986, 3104 and 1173, for the *Nc*AA9C native, the *OST1* and the pre-*OST1*-pro-α-factor signal peptide, respectively.

**Fig. 6:**
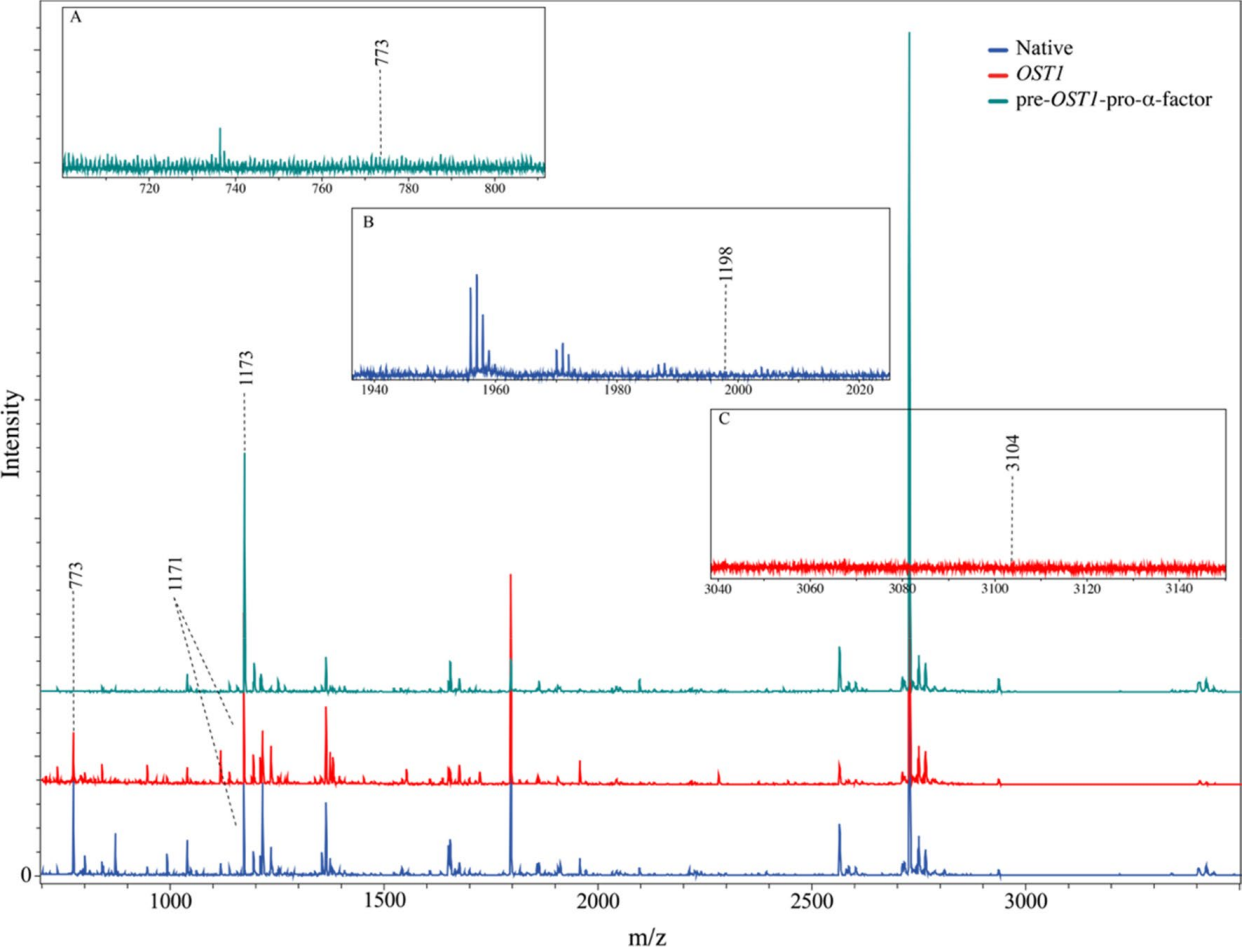
MALDI-ToF spectra of the trypsin digested *Nc*AA9C expressed with the different signal peptides. The different colors present the different constructs. The spectrum of the LPMO expressed with the native signal peptide is shown in blue whereas the enzyme expressed with *OST1* is presented in red and the one produced with the pre-*OST1*-pro-α-factor in green. The inlaid boxes show the spectra in the areas in which the masses of the correctly processed N-terminus in case of the pre-*OST1*-pro-α-factor (**A**) or the wrongly processed N-termini of the other two constructs (**B**, **C**), would be expected

In the spectra of the *Nc*AA9C samples with the native and the *OST1* signal peptide peaks at the mass of 773 could be identified (Fig. 6, red and blue trace). In both spectra, no peaks, at m/z values of 1986 and 3104 corresponding to wrongly processed N-termini could be detected (small inlaid boxes in Fig. 6). In the spectra of *Nc*AA9C samples secreted with the *OST1*-pro-α-factor, a peak at a m/z value of 1173 could be found, which corresponds to a peptide with the sequence: “EAEA**H**TIFQK” (Fig. 6, green trace). Therefore, we presume that the previously observed missing functionality of this sample is due to incorrect signal peptide cleavage, which resulted in a wrongly processed N-terminus and loss of enzyme activity. The “EAEA” found N-terminal of the terminal histidine is part of the Ste13 cleavage site, which is notoriously problematic in terms of N-terminal processing [20].

In summary, the data confirm that the *OST1* signal peptide both successfully secrets *Nc*AA9C and is correctly recognized and cleaved by *P. pastoris*, which results in a homogenously processed N-terminus and full enzymatic functionality. In contrast, the pre-*OST1*-pro-α-factor is incorrectly processed, which leads to a loss of activity of *Nc*AA9C and makes this signal peptide unsuitable for recombinant production of LPMOs. This finding is in line with reports of Tanghe et al. [18], who reported the unsuitability of the α-factor for secretion of LPMOs, as the pre-*OST1*-pro-α-factor and the native α-factor signal peptide share the same pro- sequence, which is adjacent to the N-terminus of the mature protein.

### Expanding to other LPMO families

To further investigate the applicability of the presented novel expression tools for recombinant production of LPMOs, we expanded the testing to other LPMO families. Additionally, to the already presented LPMOs *Ls*AA9A and *Nc*AA9A, we produced a single-domain AA13 from *Aspergillus oryzae* (*Ao*AA13) and a multidomain AA11 from *Aspergillus fumigatus* (*Af*AA11B) using pBSYP_*GCW14*_Z and pBSYP_*GCW14*_Z-*OST1,* respectively. For *Ao*AA13 this is, to our best knowledge, the first report of a successful recombinant expression of active enzyme using *P. pastoris* as all published articles refer to the same patent that describes the expression in *A. oryzae* [38]*.*

Expression and purification of *Ao*AA13 was done as described above. Again, expression analysis by SDS-PAGE indicated a rather clean expression broth with mainly recombinant, LPMO which allowed us to use one single SEC step for purification which yielded in pure enzyme (Fig. 2C). The enzyme is on the gel visible as a band at ~ 25 kDa which is in accordance with the expected theoretical calculated mass of 27.8 kDa (Fig. 1). For the *Ao*AA13 we got 1.58 ± 0.11 mg of protein from 50 ml expression broth corresponding to ~ 30 mg pure LPMO from 1 L culture supernatant which is comparable to the yields we obtained for other LPMOs.

For *Af*AA11B protein expression was lower than for the other tested LPMOs. To ensure complete isolation of secreted *Af*AA11B of the expression supernatant, a hydrophobic interaction chromatography (HIC) step was introduced to bind the protein on the column and elute it in a small volume to reduce the time required for concentration prior SEC. The SEC purification was executed as described for the other LPMOs, and *Af*AA11B eluted in a single peak off the column. In the subsequent SDS-PAGE analysis a double band was observed (Fig. 2C) with the LPMO at ~ 60 kDa and a contamination at ~ 20 kDa. Based on the previous in silico analysis we expected heavy glycosylation (Fig. 1) of *Af*AA11B which is confirmed by SDS-PAGE analyzes as the enzyme appears to be about 17 kDa bigger than the theoretical calculated molecular weight (42.8 kDa).

Repeating the SEC purification step with a fraction of the purified LPMO did not change the composition of the protein solution (double band on SDS gel), which indicates that the second band is not from a native *P. pastoris* protein. We presume, that the LPMO linker region with the CBM separates from the catalytic domain during the preparation of the sample prior to the SDS-PAGE analysis or in the analysis, we do not know exactly how, which results in the second protein form. From the process we obtained 1.38 ± 0.00 mg of pure protein from 500 mL cultivation broth, which is about 10 times less in comparison to the other tested LPMOs.

**Fig. 7:**
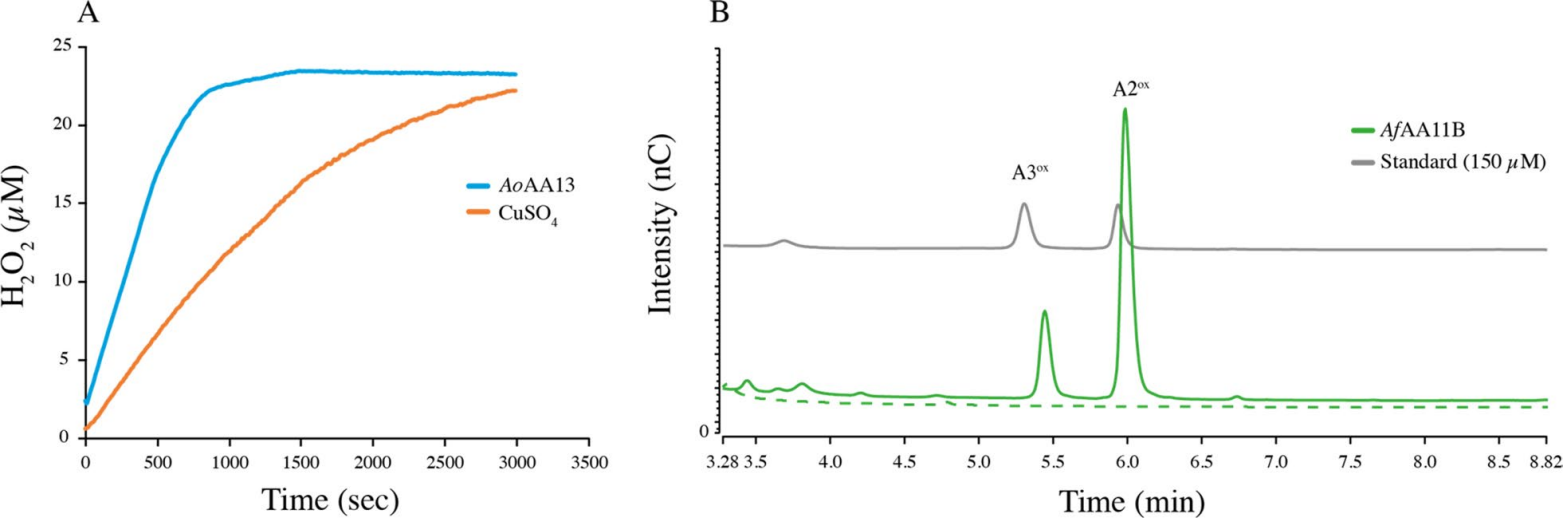
H_2_O_2_ production curve (**A**) and HPEAC-PAD profile (**B**) to confirm the functional expression of *Ao*AA13 and *Af*AA11B by using either the pBSYP_*GCW14*_Z or the pBSYP_GCW14_Z-*OST1* expression plasmid. **A** For the H_2_O_2_ production curve 3 µM *Ao*AA13/CuSO_4_, 100 µM Amplex Red and 0.025 mg/ml HRP were used, and the reaction was initiated by the addition of 50 µM AscA (all final concentrations). Control reactions did not contain catalyst. The presented curves represent the average of three independent replicates. **B** To assess the substrate oxidation ability of *Af*AA11B standard aerobic reactions (green) with 1 µM LPMO, 1 mM (GlcNAc)_4_ in the presence (solid lines) and absence (dashed lines) of 1 mM AscA were made. To show that peaks correspond to oxidized products, a standard containing 150 µM oxidized chitobiose and chitotriose is shown (grey)

The substrate oxidation ability of 1 µM *Ao*AA13 was tested by incubation with 0.1% starch, maltopentaose, panose, maltotriosyl-maltotriose or ismaltotriose (1 mM each) in the presence and absence of 1 mM reductant overnight. From none of these substrates oxidized products could be detected, which is in agreement with published results of this particular LPMO on starch [39]. To confirm functional expression of *Ao*AA13, the oxidase activity assay described by Kittl et al*.* [22] was used, which is based on the enzymes ability to produce H_2_O_2_ from a reduced copper center. To ensure that the observed H_2_O_2_ does not origin from unbound copper, control reactions with CuSO_4_ were included. From the progress curves (Fig. 7A) H_2_O_2_ production rates of 0.03 ± 0.003 μM*s^−1^ and 0.01 ± 0.001 µM*s^−1^ for the reaction with LPMO and CuSO_4_ were calculated, respectively, which confirms the structural integrity of the active site and indicates that we lack the correct carbohydrates to detect the formation of oxidized products.

To confirm the substrate oxidation ability of *Af*AA11B and therefore its functional secretion using the *OST1* as a leader sequence, the enzyme was incubated with (GlcNAc)_4_ in the presence and absence of AscA (1 mM) over night. Reaction products were analyzed by HPAEC-PAD and the chromatograms (Fig. 7B) confirm the release of oxidized products from *Af*AA11B in the presence of reductant (solid lines) and the absence of products in the control reaction without reductant (dashed lines) confirming the correct processing of the N-terminus.

## Conclusion

The presented study gives novel insights into the expression of fungal LPMOs using the industrially most relevant expression host *P. pastoris*. The presented data show the successful and active expression of four LPMOs from three different fungal LPMO families, one of which (*Ao*AA13) has, to our best knowledge, never before been expressed in a lower eukaryotic host.

The implementation of advanced molecular biology tools for the genetic manipulation of *P. pastoris* made cloning, expression and downstream processing as effortless as possible. Our data demonstrate that both tested vectors that employ differently regulating promoters (pBSY3Z and pBSYP_*GCW14*_Z) in combination with the *Pichia pastoris* strain BSYBG11 are suitable for the expression of LPMOs. The presented protocols are standardized, require little equipment, short cultivation periods and result in up to 42 mg of pure LPMO per liter of cultivation supernatant with the limited effort of a medium-scale cultivation. The use of medium-scale shake flasks with subsequent tag-less single-step purification makes the production of valuable LPMOs no longer tedious and time consuming and abolishes enzyme production as limiting factor in this field of science.

To expand the toolbox for LPMO-tailored expression further we report, for the first time, a non-LPMO-originating signal peptide that facilitates secretion of active LPMOs. The *OST1* signal peptide in combination with the presented regulatory tools, facilitates an efficient way for LPMO discovery, as cloning is streamlined, and the achieved protein concentrations are high enough to determine substrate oxidation activity from crude supernatant. Here, the *OST1* presents an interesting alternative to using an LPMOs native signal peptide, as it provides a way to express bacterial LPMOs in *P. pastoris* or to overcome potential inactivation caused by incorrectly processed N-termini.

Moreover, this is, to our best knowledge, only the fourth time the *OST1* signal peptide has been reported for secretion of proteins with *P. pastoris* and the second time the secretion of an enzyme is reported [34, 35, 40]. Additionally, we are the first ones to investigate N-terminal processing of this signal peptide by MS*.* The fact that this N-terminal leading sequence is cleaved perfectly makes it an interesting candidate for the production of proteins that require a largely homogenous N-terminus, i.e., pharmaceutically relevant proteins.

## Material and methods

### Chemicals, microorganisms and media

All chemicals were purchased from Carl Roth (Karlsruhe, Germany), VWR or Sigma-Aldrich. Oligonucleotides were ordered from Integrated DNA Technologies (Leuven, Belgium) and BioXP® regents from SGI-DNA, Inc. (San Diego, CA, USA). Kits used for plasmid isolation (Wizard® Plus SV Minipreps DNA Purification Systems) and purification of agarose gel slices, PCRs and restriction digests (Wizard® SV Gel and PCR Clean-Up System) were purchased from Promega (Fitchburg, WI, USA). For cloning by Gibson isothermal assembly, Gibson Assembly® HiFi 1-Step Kit (SGI-DNA, Inc., San Diego, CA, USA) was used, all other enzymes and Phusion DNA polymerase were obtained from Thermo Fisher Scientific (Waltham, MA, USA).

For standard cloning procedures and plasmid propagation, self-made chemically competent *Escherichia coli* XL1-Blue were used (Mix & Go! *E. coli* Transformation Kit and Buffer Set, Zymo research, Irivine, CA, USA). For amplification of BioXP synthesized plasmids, NEB® 5-alpha Competent *E. coli* (High Efficiency) cells (New England Biolabs, Ipswich, MA, USA) were used and transfection was performed according to the manual for high efficiency transformation. As an eukaryotic expression host Bisy GmbH provided the killer plasmid-free *P. pastoris* strain BSYBG11(Δ*AOX1*, Mut^S^), which originates from BG08 (BioGrammatics Inc., Carlsbad, CA, USA) and is a NRRL Y‐11,430 derivative [41].

*E. coli* strains were cultivated in/on LB-medium (Luria/Miller) supplemented with Zeocin to a final concentration of 25 μg/mL (Life Technologies, Carlsbad, CA, USA).

For selection and expression of *P. pastoris* strains YPD containing 1% (w/v) yeast extract, 2% (w/v) peptone and 2% (w/v) glucose) was used. For selection following transformation and re-streaking of transformants and expression clones YPD agar plates were supplemented with100 μg/ml Zeocin.

### Assembly of the novel vector backbone

**Fig. 8:**
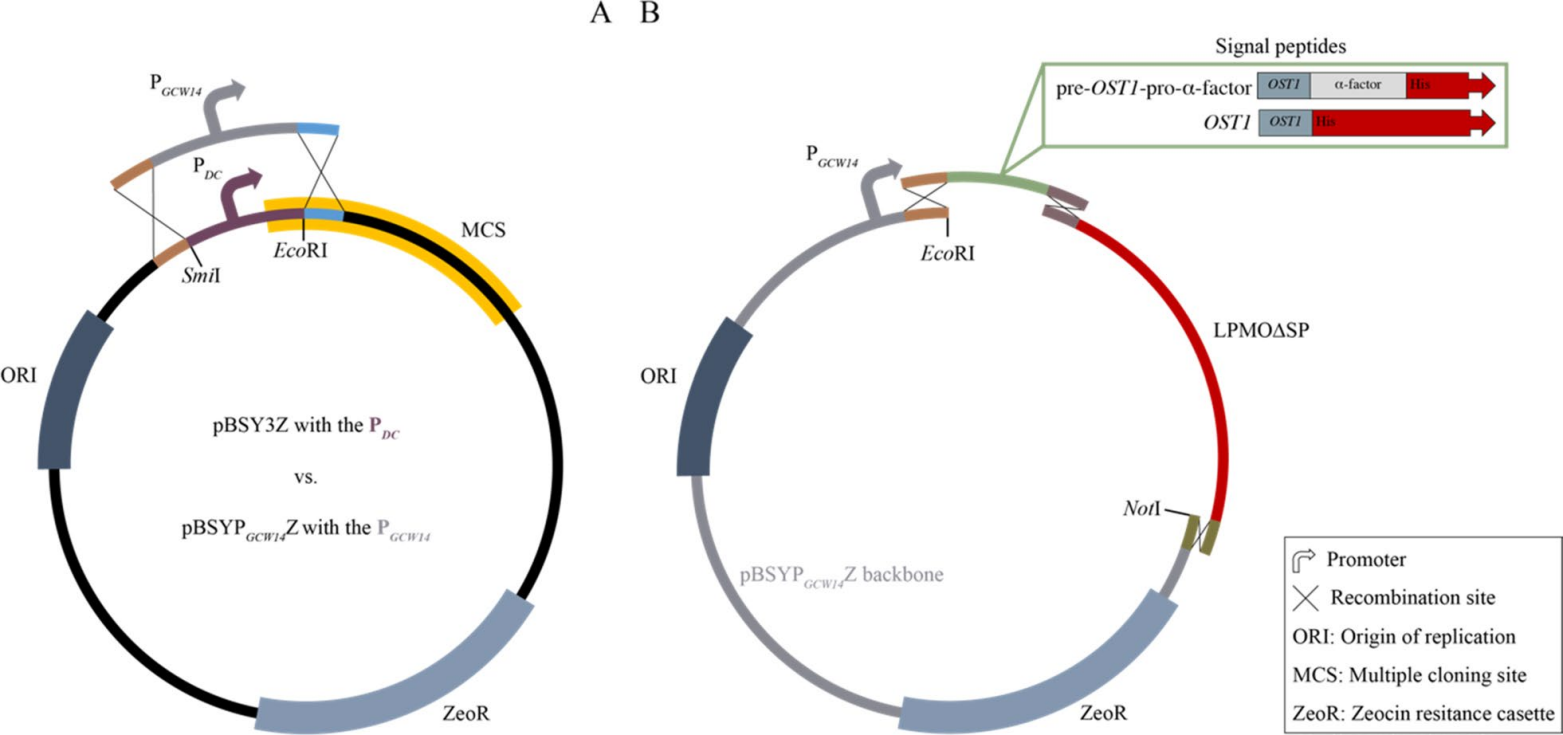
Schematic overview on the manually performed cloning. **A** Assembly of the new pBSYP_*GCW14*_Z expression plasmid by exchanging the P_*DC*_ promoter of the commercially available pBSY3Z plasmid controlling the transcription of the GOI against the P_*GCW14*_ promoter. **B** Cloning strategy used for the assembly of the expression plasmids with the alternative SP by homologues recombination cloning. The pBSYP_*GCW14*_Z plasmid was used as backbone to insert the PCR-amplified yeast-originating *OST1* or the artificial pre-*OST1*-pro-α-factor and a LPMO gene of which the natural SP had been removed (LPMO∆SP) by PCR

Throughout this study, we used the commercially available *P. pastoris/E. coli* shuttle vector pBSY3Z (Bisy GmbH, Hofstätten a. d. Raab, Austria) and the newly assembled pBSYP_*GCW14*_Z. The pBSYP_*GCW14*_Z plasmid is except of the promotor that controls the expression of the GOI identical to the pBSY3Z plasmid. To exchange the promoter (P_*DC*_), we digested the pBSY3Z plasmid with *Eco*RI and *Smi*I and inserted the PCR-amplified P_*GCW14*_ promoter [25, 41] that contained 5´ and 3´ homologous regions to the vector backbone by Gibson assembly® upstream of the multiple cloning site (MCS) (Fig. 8A). The sequence of the newly assembled pBSYP_*GCW14*_Z was verified by Sanger sequencing (Microsynth AG, Balgach, Switzerland).

### LPMO construct assembly

Molecular biological in silico design of the expression plasmids was performed using SnapGene (Chicago, IL, USA). De novo synthesis of the LPMO genes and cloning were performed using the BioXP 3000 system (SGI-DNA, Inc., San Diego, CA, USA). Since the BioXP-mediated cloning is based on homologous recombination cloning, the 5´ and 3´-ends of the codon-optimized genes of *Ls*AA9A (AN: ALN96977), *Nc*AA9C (AN: XP_965598), *Af*AA11B (AN: XP_748042) and *Ao*AA13 (AN: XP_023092053) that included the native LPMO SP were designed to have a 30 bp overhang to the promoter and terminator region of the target vector backbone. The BioXP 3000 system was set up according to the manual and the cloning strip of the instrument was filled with 23 µl of 20 ng/µl *Sap*I linearized pBSY3Z and pBSYP_*GCW14*_Z, respectively. The output of the BioXP run was in total eight plasmids with either the pBYSP_*GCW14*_Z or the pBYS3Z expression plasmid into which one of the four LPMO genes had been cloned.

Expression plasmids with the alternative SP are based on the pBYSP_*GCW14*_Z backbone. Assembly was done using three DNA fragments, one was the linearized vector backbone pBYSP_*GCW14*_Z, one the respective LPMO without its native signal sequence and the last one the novel alternative signal sequence. The native signal peptides of *Nc*LPMO9C and *Af*AA11B were removed by PCR employing primer pair one or two (Table 4) using the newly synthesized LPMO expression plasmids as template resulting in *Nc*AA9C∆SP and *Af*AA11B∆SP, which are for now on referred to as LPMO∆SP. The *OST1* and pre-*OST1*-pro-α-factor signal sequence (SP) (Table 3) were amplified from the commercially available plasmid pBSY3S2Z (Bisy GmbH, Hofstätten a. d. Raab, Austria) which contains the pre-*OST1*-pro-α-factor by using primer pair three and four, respectively (Table 4). Since our cloning strategy is based on homologous recombination cloning, we introduced 5´ and 3´ overhangs to the flanking DNA regions during amplification. This means that the 5´-ends of the PCR-amplified SP were overlapping with the promoter region of the pBSYP_*GCW14*_Z plasmid and the 3´-end overlapped with the 5´-end of the LPMO∆SP genes. Similarly, the PCR-amplified LPMO∆SP gene had a homologous region to the SP fragment at the 5´-end and an overhang to terminator region of the plasmid backbone at the 3´-end. Prior to Gibson isothermal assembly the pBSYP_*GCW14*_Z plasmid was digested with *Not*I and *Eco*RI which resulted in a linear DNA fragment that contained the required homologous region to the signal peptide fragment at the promotor region and the correct overlap to the LPMO∆SP fragment at the terminator region.

**Table 3:** DNA sequence of the tested yeast-originating *OST1* and the artificial pre-*OST1*-pro-α-factor secretion signal

Signal peptide	DNA sequence 5` 3`
*OST1*	atgaggcaggtttggttctcttggattgtgggattgttcctatgttttttcaacgtgtcttctgct
pre-*OST1*-pro-α-factor	atgaggcaggtttggttctcttggattgtgggattgttcctatgttttttcaacgtgtcttctgctgcccctgttaacactaccactgaagacgagactgctcaaattccagctgaagcagttatcggttactctgaccttgagggtgatttcgacgtcgctgttttgcctttctctgcttccattgctgctaaggaagagggtgtctctctcgagaagagagaggccgaagct

**Table 4:** Primer pairs used for DNA amplification

#	Construct	DNA sequence 5`→3`
1	*Nc*AA9C∆SP	
	FWD	catacaatttttcagaaggtgtcagtcaacg
	REV	tggcattctgacatcctcttgagc
2	*Af*AA11B∆SP	
	FWD	catatgaagatgagacaacccactccatattc
	REV	tggcattctgacatcctcttgagc
3	*OST1*	
	FWD	gtcactcgcttcactcaacaacaaaaatgaggcaggtttggttctcttgg
	REV	ccgttgactgacaccttctgaaaaattgtatgagcagaagacacgttgaaaaaacataggaac
4	pre- *OST1*-pro-α-factor	
	FWD	gtcactcgcttcactcaacaacaaaaatgaggcaggtttggttctcttgg
	REV	ccgttgactgacaccttctgaaaaattgtatgagcttcggcctctctcttctcg

For the final plasmid construction, we followed the manual provided with the Gibson assembly® kit. The pBSYP_*GCW14*_Z-*OST1*-*Nc*AA9C construct was assembled by incubation of the linearized pBSYP_*GCW14*_Z backbone with the *OST1* and *Nc*AA9C∆SP fragment. The pBSYP_*GCW14*_Z-pre-*OST1*-pro-α-factor-*Nc*AA9C and the pBSYP_*GCW14*_Z-*OST1*-*Af*AA11B constructs were assembled identically but instead of the *OST1* we used the pre-*OST1*-pro-α-factor fragment and instead of the NcAA9C∆SP we used the *Af*AA11B∆SP fragment, respectively (Fig. 8B). Sequence of the plasmids was verified by Sanger sequencing (Microsynth AG, Balgach, Switzerland).

### Pichia pastoris transformation and screening

The expression plasmids were *Smi*I linearized prior to transformation of the eukaryotic host. Transformation of the *Pichia pastoris* BSYBG11 one-shot ready competent cells (Bisy GmbH, Hofstätten a. d. Raab, Austria) was done according BSY *Pichia pastoris* transformation protocol.

Following 48 h of growth and antibiotic selection on agar plates, 24 transformants of each construct were randomly selected for expression analysis. Microscale cultivation (96 deep-well plate cultures) of *P. pastoris* cells carrying constructs based on pBSY3Z was done according to the BSY high-throughput screening protocol, which is based on the work of R. Weis et al*.* [42]. Microscale cultivation (96 deep-well plate cultures) of *P. pastoris* cells carrying constructs based on pBSYP_*GCW14*_Z was performed in YPD media over 60 h due to the P_*GCW14*_, which facilitates methanol-independent constitutive expression.

At the end of the cultivation, optical cell density of each microscale culture was determined as absorbance at 600 nm (OD_600_). Additionally, total protein amount secreted by each culture was determined by Bradford protein assay after harvesting the culture medium by centrifugation at 4000 rpm, 4 °C for 10 min. For each culture, we normalized the protein amount by the corresponding OD_600_ and corrected by the wild-type secretion. This allowed us to select the highest secreting clone for further characterization and evaluate the average expression for each construct. Additionally, expression of the target protein by selected clones was confirmed by sodium dodecyl sulphate–polyacrylamide gel electrophoresis (SDS-PAGE). If not mentioned different protein samples were reduced and denatured prior SDS-PAGE analysis.

### Expression, purification, and copper loading

500 ml YPD (1% (w/v) yeast extract, 2% (w/v) peptone and 2% (w/v) glucose) in a 2-L baffled shake flask was inoculated with a fresh single yeast colony and incubated for 60 h at 28 °C and 120 rpm. Cells and supernatant were separated by centrifugation at 10,000x*g* and 4 °C for 15 min. The protein containing supernatant was filtered by a 0.22 µM Steritop® bottle-top filter (Merck Millipore, Burlington, MA, USA) and concentrated fivefold by a VivaFlow 200 tangential crossflow concentrator (molecular weight cut-off, MWCO 10 kDa, Sartorius Stedim Biotech Gmbh, Germany).

For size exclusion chromatography (SEC), we used a HiLoad 16/60 Superdex 75 size exclusion column (GE Healthcare Life Sciences, Uppsala, Sweden) that was equilibrated with 50 mM BisTris/HCl buffer (pH 6.5) containing 150 mM NaCl and operated with an Äkta purifier (GE Healthcare Life Sciences, Uppsala, Sweden). Prior to loading of the samples onto the column, the expression supernatant was concentrated tenfold. We typically applied small volumes (< 1 mL) to column to ensure good separation and the isolation of pure protein. The protein was eluted and fractionated using a flow rate of 1 ml/min. Fractions containing the pure enzyme were identified using SDS-PAGE, pooled, and concentrated using Amicon Ultra centrifugal filters (MWCO 10 kDa, Merck Millipore, Burlington, MA, USA).

When a hydropic interaction chromatography (HIC) step was performed prior SEC, we added ammonium sulfate to a final concentration of 2.4 M to the protein containing supernatant. Here, we used a 5 mL HiTrap Phenyl FF column (GE Healthcare Life Sciences, Uppsala, Sweden) equilibrated with 50 mM BisTris/HCl buffer (pH 6.5), containing 2.4 M ammonium sulfate. To elute the protein from the column, a 35 ml linear gradient from 2.4 M to 0 M ammonium sulfate in 50 mM BisTris/HCl buffer (pH 6.5) using a flowrate of 1.8 ml/min was used. The collected fractions were analyzed by SDS-PAGE and pooled if the target protein was present. Prior to isolation by SEC, the protein containing solution was concentrated using Amicon Ultra centrifugal filters (MWCO 10 kDa, Merck Millipore, Burlington, MA, USA).

Copper saturation of the purified LPMO was ensured by adding a 1:3 molecular ratio of enzyme to CuSO_4_ and left for incubation for 1 h at *t* = 4 °C. Remaining copper and salt were removed by exchanging the total volume to 50 mM BisTris/HCl buffer (pH 6.5) four times using Amicon Ultra centrifugal filters (MWCO 10 kDa, Merck Millipore, Burlington, MA, USA). The homogeneity of the enzymes was evaluated by SDS-PAGE and concentration was measured by the Bradford assay. The purified and copper-saturated proteins were stored at 4 °C until further use.

### LPMO reactions

Standard LPMO reactions contained 1-5 µM enzyme, 0.1% phosphoric acid-swollen cellulose (PASC) or 1 mM oligomeric substrates (95% purity; Megazyme, Wicklow, Ireland) in 50 mM BisTris/HCl buffer (pH 6.5) and were incubated at *t* = 37 °C and 750 rpm (Thermomixer C, Eppendorf, Hamburg, Germany). Reactions were initiated by the addition of 1 mM AscA and either terminated by boiling (crystalline substrates) or by the addition of 7 reaction volumes of 200 mM NaOH (soluble substrates).

Reaction products were detected using high‐performance anion exchange chromatography with pulsed amperometric detection (HPAEC‐PAD). For the analyses, a Dionex ICS5000 system, equipped with a CarboPac PA200 analytical column and a CarboPac PA200 guard column, with a 26 min gradient for cellulose and an 18 min gradient for chitin containing samples [43].

The activity of the cellulose-active LPMOs was assessed by quantification of the native products that would proportionally increase upon oxidation of soluble substrates. For the confirmation of oxidized chitin products, in-house made standards were used as described elsewhere [44]. Chromatograms were recorded and analyzed with Chromeleon.

### H_2_O_2_ production assay

The formation of H_2_O_2_ was measured to determine the LPMOs oxidase activity as described previously by Kittl et al. [22]. The reaction was carried out in 50 mM BisTris/HCl buffer (pH 6.5) and contained 3 µM LPMO, 100 µM Amplex Red and 0.025 mg/ml HRP. The reaction was initiated by the addition of AscA (final concentration 50 µM) after 5 min preincubation at *t* = 30 °C. The formation of resorufin was monitored at 540 nm. Blank reaction did not contain LPMO and standards had AscA added to capture potential interactions between the substrate/product and the reductant.

### MALDI-ToF analysis

Matrix-assisted laser desorption/ionization time of flight mass spectrometry (MALDI-ToF MS) analyses were performed on an Ultraflex MALDI‐ToF/ToF instrument (Bruker Daltonik GmbH, Bremen, Germany) equipped with a Nitrogen 337 nm laser applying suitable preinstalled instrument methods.

To investigate the homogeneity of our protein stocks, the purified protein was mixed in a 1:1 ratio with a 2% TFA solution. As matrix a solution containing 3.8 mg/ml 2,5-DHAP and 4.5 mg/ml DAC in 75% ethanol was used. Prior analysis the protein solution and matrix were mixed in a 1:1 ratio and spotted on a ground steel plate.

The correct processing of the N-terminus was examined by trypsin fragmentation of the LPMOs with subsequent MALDI-ToF analysis. MS sample preparation followed the protocol described by Tuveng et al. [45]. For reduction and acetylation of the protein sample, 30 µg of the purified enzyme was mixed with dithiothreitol (DTT, final concentration 10 mM) in Eppendorf LoBind tubes and incubated for 30 min. Subsequently, indole-3-acetic acid (IAA, final concentration of 15 mM) was added and incubated another 30 min in the dark before digestion of the enzymes with 0.75 µg trypsin (Sequencing Grade Modified Trypsin, Promega) over night at 37 °C. The reaction was quenched with trifluoroacetic acid (TFA, final concentration of 1%) and concentrated by C_18_ solid phase extraction ZipTips (Merck Millipore, Cork, Ireland). The peptides were eluted in 10 µl 70% ACN with 0.1% TFA and dried under vacuum. The peptide pellet was suspended in 10 µL of 2% ACN with 0.1% TFA. Prior to analysis, the protein samples, and the matrix (HCCA saturated in TA30) were mixed in a 1:1 ratio and spotted on a ground steel plate. The peptide mass lists (Table 5) for the different LPMO constructs were generated using the ExPASy online tool PeptideMass (https://web.expasy.org/peptide_mass/). The peptides containing the N-terminal histidine are highlighted in bolt.

**Table 5:** List with the theoretical peptide masses of the trypsin digested *Nc*AA9C expressed with the different signal peptides

Fragment	Masses expected if the signal peptide is cleaved correctly	Masses expected of a wrongly processed N-terminus		
	Independent of SP	Native	*OST1*	pre- *OST1*-pro-α-factor
1	**773.4304**	**1986.1018**	**3104.5855**	6754.3264
2	1215.6328	1215.6328	1215.6328	890.4465
3	2505.1782	2505.1782	2505.1782	**1173.5898**
4	1171.6317	1171.6317	1171.6317	1215.6328
5	2725.2865	2725.2865	2725.2865	2505.1782
6	991.5645	991.5645	991.5645	1171.6317
7	1234.6273	1234.6273	1234.6273	2725.2865
8	945.4999	945.4999	945.4999	991.5645
9	3347.5322	3347.5322	3347.5322	1234.6273
10	5926.898	5926.898	5926.898	945.4999
11	6678.2651	6678.2651	6678.2651	3347.5322
12	1020.5142	1020.5142	1020.5142	5926.898
13	1469.7091	1469.7091	1469.7091	6678.2651
14	1038.5037	1038.5037	1038.5037	1020.5142
15	1355.5718	1355.5718	1355.5718	1469.7091
16	1359.5674	1359.5674	1359.5674	1038.5037
17				1355.5718
18				1359.5674

The second column shows the expected masses of a correctly processed enzyme and is therefore independent of the leading sequence. In the columns 3 to 5 the expected masses of wrongly processed N-termini of the different signal peptides are listed. Highlighted in bold are the masses of the peptides containing the N-terminal histidine

## Data Availability

All data generated or analyzed during this study are included in this published article.
